# gapFinisher: A reliable gap filling pipeline for SSPACE-LongRead scaffolder output

**DOI:** 10.1371/journal.pone.0216885

**Published:** 2019-09-09

**Authors:** Juhana I. Kammonen, Olli-Pekka Smolander, Lars Paulin, Pedro A. B. Pereira, Pia Laine, Patrik Koskinen, Jukka Jernvall, Petri Auvinen

**Affiliations:** 1 DNA Sequencing and Genomics Laboratory, Institute of Biotechnology, University of Helsinki, Helsinki, Finland; 2 Department of Neurology, Helsinki University Hospital, Helsinki, Finland; 3 Evolutionary Phenomics Group, Institute of Biotechnology, University of Helsinki, Helsinki, Finland; University of California Los Angeles, UNITED STATES

## Abstract

Unknown sequences, or gaps, are present in many published genomes across public databases. Gap filling is an important finishing step in *de novo* genome assembly, especially in large genomes. The gap filling problem is nontrivial and while there are many computational tools partially solving the problem, several have shortcomings as to the reliability and correctness of the output, i.e. the gap filled draft genome. SSPACE-LongRead is a scaffolding tool that utilizes long reads from multiple third-generation sequencing platforms in finding links between contigs and combining them. The long reads potentially contain sequence information to fill the gaps created in the scaffolding, but SSPACE-LongRead currently lacks this functionality. We present an automated pipeline called gapFinisher to process SSPACE-LongRead output to fill gaps after the scaffolding. gapFinisher is based on the controlled use of a previously published gap filling tool FGAP and works on all standard Linux/UNIX command lines. We compare the performance of gapFinisher against two other published gap filling tools PBJelly and GMcloser. We conclude that gapFinisher can fill gaps in draft genomes quickly and reliably. In addition, the serial design of gapFinisher makes it scale well from prokaryote genomes to larger genomes with no increase in the computational footprint.

## Introduction

Gap filling is one of the final phases of genome assembly, especially in large genomes. First, assembly algorithms produce contiguous sequences of overlapping sequencing reads known as contigs. A contig is a continuous DNA sequence entity without any ambiguities or unknown bases marked as N. Second, the contigs are connected into longer fragments using specialized sequencing read data in a process called scaffolding. Until the development of long read technologies, the data for scaffolding used to be primarily mate-pair reads. The mate-pair libraries sometimes also called jumping libraries [1], are usually made of size selected DNA fragments, where the fragment size is usually in the order of thousands of base pairs. The ends of the fragments are then sequenced, and the resulting reads are used for creating links between the contigs. The linked sequences are known as scaffolds, and the unknown sequence between the contigs is commonly marked with N-characters. Currently, long continuous reads e.g. from Pacific Biosciences RS II or Sequel third-generation sequencing platforms are commonly used in scaffolding. While the scaffolding step links and orders the contigs, it usually leaves variable amounts of unknown sequences in the final product. These unknown sequences are called gaps. Finally, the gap filling stage aims to resolve these unknown sequences with [2,3] or without [4] additional sequencing data. Even with the gap filling step applied, substantial gaps do exist in many published genomes. Examples include the *Mus musculus* (house mouse, 78,088,216 base pairs gaps) and *Mustela putorius furo* (ferret, 132,851,443 base pairs gaps) chromosome level assemblies in the ENSEMBL database [5].

In this study, we present an automated gap filling pipeline called gapFinisher. We pursue a solution to the gap filling problem that utilizes long reads and unaltered draft genomes. We set strict alignment parameters for the gap filling stage to ensure correctness and uniqueness of the filled gaps. In addition, we benchmark the performance of gapFinisher against two published gap filling tools PBJelly [6] and GMcloser [7]. We selected PBJelly and GMcloser for the benchmark because of their popularity and ability to process long-read data. We conclude that applying gapFinisher enables efficient and reliable gap filling by controlling the use of the FGAP algorithm [8]. Furthermore, gapFinisher computing times prove linear with respect to the size of the input.

### From scaffolding to gap filling

SSPACE-Standard [2] and SSPACE-LongRead (SSPACE-LR) [9] are scaffolding tools for paired-end (also mate-pair) reads and long continuous reads, respectively. While these tools are available free for academic users, both are commercial products, and upgrades and most of the support require a proprietary license. SSPACE-Standard is commonly applied in the first scaffolding steps where contigs are oriented and ordered into the initial longer connected sequences. SSPACE-Standard accepts paired-end data from any next-generation sequencing technology if read-orientation information and mean values and standard deviations of the insert sizes for each read library are provided [2]. SSPACE-LR utilizes Pacific Biosciences filtered subreads (CLR = *Continuous Long Reads*) in finding even longer links between contigs or existing scaffolds and combining them into “superscaffolds” with new gaps introduced between the sequences [9]. SSPACE-LR first maps the long reads into the contig assembly using the BLASR aligner specialized for long read alignment [10]. Based on these alignments, contigs are then linked into scaffolds and N-characters (gaps) are placed between the connected contigs. While the CLR reads contain information of the actual nucleotide sequence in the gaps, this feature is not exploited in the current version of SSPACE-LR (version 1.1). However, the software can report the exact information about which reads were associated when creating the new scaffold and the new gap(s). In the gapFinisher pipeline, we utilize this information to fill the gaps in the newly created scaffolds on the go.

A central part of gap filling is the alignment of long sequences against the contigs. This is challenging due to the relatively high error-rates of contemporary long read data [11] and the sequencing errors [12,13] and local misassemblies at the contig level [9]. The BLAST local alignment tool [14] is the most commonly used approach for the identification of areas of high similarity between multiple sequences. Different scaffolding and gap filling tools apply BLAST either directly [8], or the method is refined [10] and applied [6,9]. All tools based on BLAST contain multiple parameters, e.g. for mismatches and gaps, affecting their ability to detect non-perfect matches and it is not always clear how these should be defined.

Several gap filling software tools for short read data exist. GapFiller is a commercial program by the authors of the SSPACE-tools [2,9] and is often used with them [3]. GapFiller uses paired-end read information to fill in sequences at contig ends where overlapping reads reach into the gap created on the SSPACE-Standard step by mate-pair reads. Where mate-pair links do not span the whole length of the unknown sequence, the gap is not filled and unknown bases (N-characters) will remain in the output version of the draft genome [2]. Gap2Seq [15] is another gap filling tool and provides a purely computational solution to the gap filling problem for short-read data. Gap2Seq works well on most prokaryote genomes but does not scale to larger genomes, where repetitive sequences confuse the algorithm and the sheer size of the genome makes running times infeasibly long [15].

### Long-read based gap filling

There are multiple gap filling tools for long read data available today. PBJelly [6] is a scaffolding and gap filling tool integrated into the Pacific Biosciences (PacBio) SMRT Analysis software suite, the main user interface for data analysis using PacBio long reads. In comparison to other gap filling tools, the PBJelly pipeline is run in six separate stages (setup, mapping, support, extraction, assembly and output) and requires additional software libraries, preferably the SMRT Portal software suite and BLASR [10]. Although it is possible to run PBJelly in a single-core computer, the workflow is clearly designed for high-throughput computing in a grid where an additional level of automation is available, e.g. the Sun Grid Engine [16]. The single-core user is required to construct a short XML script to operate PBJelly. The six steps of PBJelly could be further automated with additional scripting. A peculiar feature of PBJelly is that it by default inflates short gaps (< 25 bp) to a length of exactly 25 bp with the apparent purpose of emphasizing the location of the gaps [6].

GMcloser [7] provides a likelihood-based approach and is suitable to both short read and long read datasets, or even more sophisticated sequence datasets to fill gaps, such as pre-assembled contigs. With GMcloser, the requirements are that user installs a Perl [17] interpreter, MUMmer [18], Bowtie2 [19] and YASS [20]. The authors of GMcloser state that their software performs better when applied multiple times to the same draft assembly with the same read data [7]. Thus, the default setting of GMcloser is to perform three iterations of gap filling in a single run.

**Fig 1:**
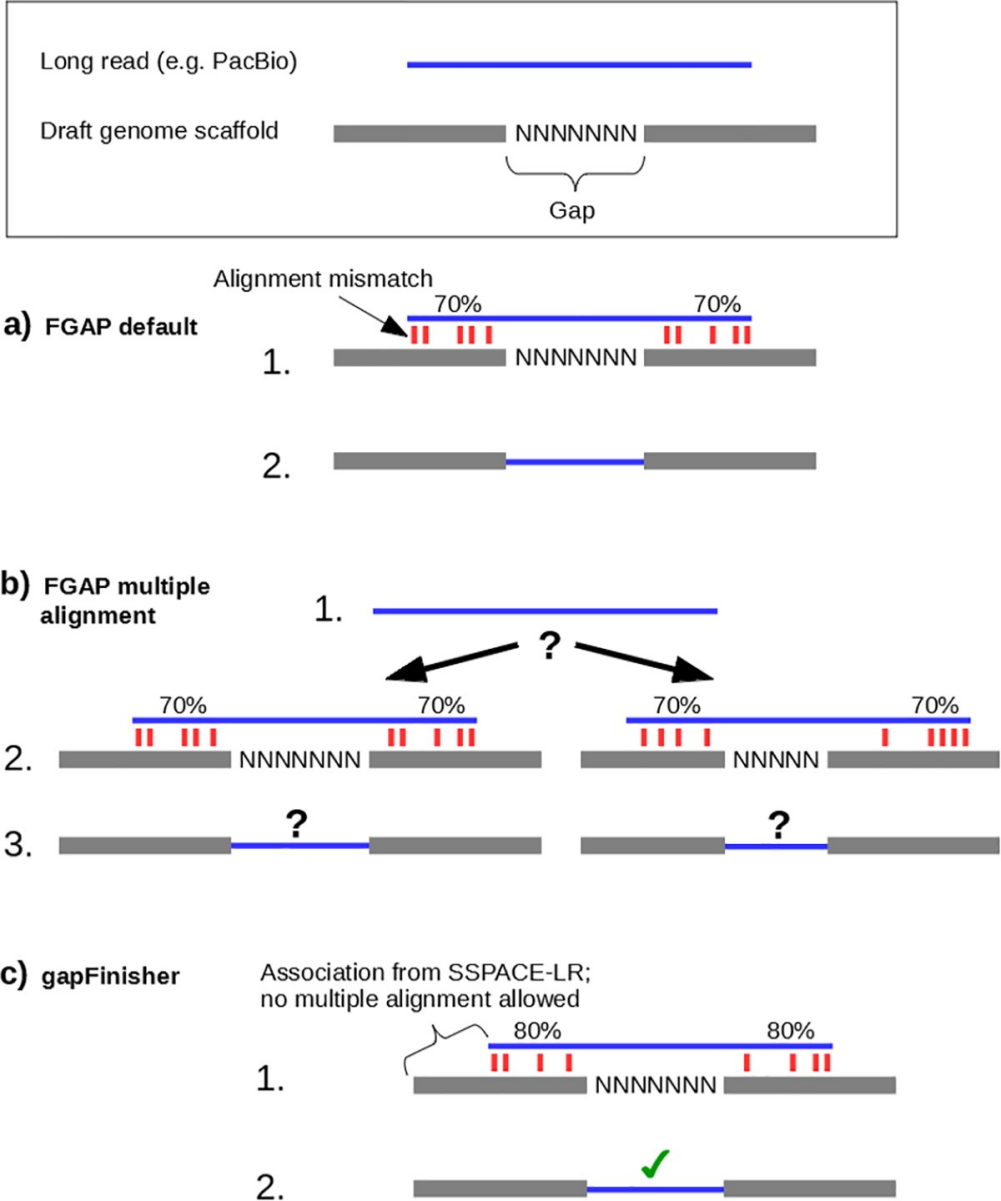
Visualization of the FGAP [8] and gapFinisher workflows. **a)** FGAP is expected to fill the gap (N-characters) between two contigs (gray blocks) using a long read (blue bar). **b)** FGAP allows the alignment of a single long read into multiple places in the genome. Ideally, a single read should align into a single location in the genome. **c)** gapFinisher uses the association of the long read and the scaffold reported by BLASR [10] used by SSPACE-LongRead [9] and ensures that each long read is only used once in gap filling.

FGAP [8] is a gap filling tool that utilizes various types of read data and BLAST alignments to find and fill gaps in draft genomes. The BLAST utility is bundled with the release version of FGAP, but a MATLAB Compiler Runtime is required. Although FGAP efficiently reduces the number of gaps in various draft genomes [8], the tool sets no limit to the number of times an input read is used in gap filling should the BLAST alignment return multiple good hits (Fig 1). With the default setting of FGAP, undesired multiple alignments of query sequences may occur due to repetitive regions in the draft genome, or overly lenient alignment parameters for the ends of the query sequences (Fig 1). We could verify this behaviour on an FGAP test run with an unpublished preliminary draft genome of a marine mammal from the *Phocidae* family (S2 Table). Ideally, gap filling should be a unique process in the sense that a single input long read should find a unique good alignment in the draft genome and fill the gaps in that single location. The gapFinisher pipeline presented in this paper is based on FGAP and enables more reliable and controlled gap filling.

Repeat masking, i.e. marking repetitive sequences in the draft genome as gaps, may improve the scaffolding and gap filling of highly repetitive draft genomes. For example, it has been estimated that more than 60% of the 3.3 Gb modern human (*H*. *sapiens*) genome consists of repetitive sequences [21]. With the repetitive sequences often found at the contig ends eliminated, the alignment algorithms are less likely to make incorrect alignments. One example of repeat masking software tools is RepeatMasker [22] which finds short and long interspersed elements as well as simple repeats in the input genomic sequence. RepeatMasker may mask coding regions of the input genome, especially those located at the terminal regions of open reading frames. Furthermore, RepeatMasker may mask some shorter potential element-coding sequences such as ribosomal RNAs [22]. While repeat masking may lower the inherent risk of incorrect alignments in specific regions, we pursue a solution that utilizes only unaltered (unmasked) draft genomes to prevent any loss of data.

Solving short gaps of e.g. 1–20 base pairs in length by simple read alignment maps produced by e.g. the Burrows-Wheeler Aligner [23] or the Bowtie 2 aligner [19] is not investigated in detail in this study but may be one of the prospects of solving the gap filling problem for short gaps. For instance, some singular unknown bases and short N-sequences at gap edges are solved by the re-assembly stage of the Pilon assembly polishing tool, where an alignment map file can be supplied as input and a specific option set for gap filling [24].

The rest of this paper is organized as follows: First, we describe the computational tools and methods we use to perform gap filling. Second, we present the example datasets for this study, namely high-throughput sequence data from six bacterial organisms and one eukaryote organism. Third, we document the results of the gap filling for the example datasets as well as the outcome of a performance benchmark of gapFinisher. Finally, we discuss the results as well as the advantages and shortcomings of the methods used.

## Materials and methods

**Fig 2:**
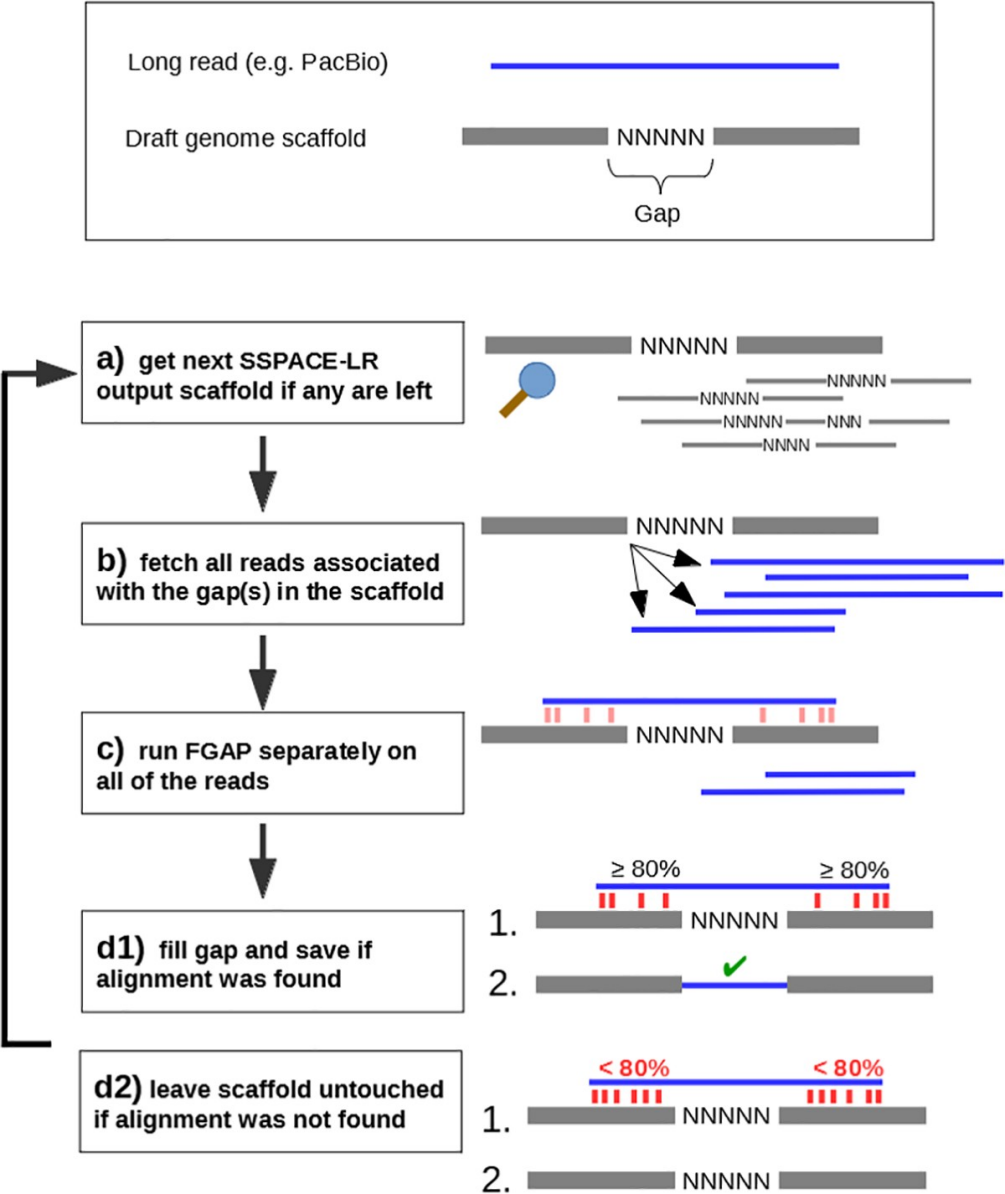
A more detailed visualization of the gapFinisher pipeline workflow. **a)** SSPACE-LR [9] reports the new scaffolds and these are iterated through one scaffold at a time. **b)** SSPACE-LR output shows the PacBio reads associated with the gaps in the scaffolds. **c)** These reads are then circulated through the FGAP [8] pipeline with only the single scaffold as input data. This logical step prevents same PacBio reads from being used in parts of the draft genome other than the current scaffold. Measures are then taken to either **d1)** replace the unknown sequence with that of the long read **(=** fill gap) or **d2)** reject the alignment and leave the gap to the genome as is.

The current release of gapFinisher works only on the output of SSPACE-LongRead [9]. The system requirements are a UNIX/Linux -based operating system, MATLAB Compiler Runtime (MCR) for FGAP and a Perl [17] interpreter for SSPACE-LR. Besides these, the gapFinisher pipeline does not require the user to install any additional software. The basic workflow of gapFinisher is illustrated in Fig 1C and in further detail below (Fig 2). Before running gapFinisher, the user must successfully run SSPACE-LR for a dataset at least once. It is imperative to have the “-k” option enabled when running SSPACE-LR. This setting will create the critical “inner-scaffold-sequences” subdirectory that contains for each superscaffold the references to the actual long read sequences (one or more) that created the scaffold. The gapFinisher pipeline will not run if this directory does not exist. When successful, gapFinisher then works as follows (Fig 2):

1. Index the draft genome FASTA file and the long read FASTA file
2. Generate a list of names of all superscaffolds SSPACE-LR (-k 1 option enabled) has created
3. For each superscaffold in the list:
  - Create a new FGAP working directory for the current superscaffold
  - Fetch all full CLR reads associated with the current superscaffold
  - For each of the CLR reads associated with the current superscaffold:
    - Execute FGAP using the current superscaffold as draft and the CLR read as input
    - If FGAP filled (one or more) gaps in the current superscaffold, save FGAP output as the new draft for the current superscaffold
4. Compile results from each working directory as filled_scaffolds.fasta
5. Compile filled_scaffolds.fasta and the unfilled/untouched scaffolds from the original draft genome as scaffolds_gapfilled_FINAL.fasta
6. [optional] Clean the working directories (to save disk space).

The rapid fetching of reads is based on the operation of the fastaindex (step 1 above) and fastafetch (step 2b above) utilities of the exonerate toolkit [25] v. 2.4.0. Pre-compiled and portable executables of these utilities are bundled with the gapFinisher release and fully integrated into the workflow of the gapFinisher pipeline.

When using PacBio filtered subreads with SSPACE-LR separate reads originating from the same well of the PacBio SMRT cell could be aligned into separate places by the BLASR aligner (Fig 1A and Fig 1B). Filtered subreads from the same well of the SMRT cell always originate from the same molecule and thus should align to locations close to one another. The legacy BLASR [10] version that SSPACE-LR is using has no formal assertion for this. Hence, we set gapFinisher to keep track of the origins of the filtered subreads. This information is contained in the FASTA headers. The pipeline issues an appropriate warning when gap filling under conflicting read origin is about to happen and aborts the filling of the gap in question. Conflicting read origins further indicate potential errors in the scaffolding step. Consequently, the location and read information of the conflict are included in the warning message and logged.

In this study, we subjected seven separate genomic sequencing read datasets from both bacterial and eukaryote organisms (Table 1) to *de novo* assembly and scaffolding. Finally, we performed gap filling on the created scaffolds with gapFinisher (Table 2). First, we had two *Escherichia coli* (*E*. *coli*) bacterial genome drafts. Second, we extended the analysis to a set of further four bacterial genomes: *Bibersteinia trehalosi*, *Mannheimia haemolytica*, *Francisella tularensis* and *Salmonella enterica*. The bacterial read data are the same that were used as test data for the SSPACE-LongRead scaffolder [9] and are available at: http://www.cbcb.umd.edu/software/PBcR/closure/index.html and the Sequencing Read Archive (SRA) links therein. For *B*. *trehalosi*, we used the reference sequence *Bibersteinia trehalosi* USDA-ARS-USMARC-188 [26]. A reference genome was available to *M*. *haemolytica* [27], although unavailable at the time of the publication of SSPACE-LongRead [9]. Finally, to get a reference on how gapFinisher performs on a much larger genome, we included an in-house unpublished marine mammal (*Phocidae* family) draft genome in final stage with 236,592 contigs scaffolded into 10,143 superscaffolds with gaps. The raw sequencing coverage of the mammal draft genome was on average 25X for the Illumina short reads and 50X for the PacBio CLR reads (Table 1). When assembled with the miniasm [28] using all the PacBio reads, we got an additional “PacBio-only” assembled version of the draft genome with 1,314 contigs which we then scaffolded into 1,115 superscaffolds and gap filled (Table 2).

**Table 1:** Next-generation sequencing read statistics and sequencing coverage for the sample datasets.

	Illumina MiSeq reads			PacBio RS reads (200X)		
Organism	Total reads	Total bases	Avg. read length	Total reads	Total bases	Avg. read length
*E*. *coli K12 MG1655*	3,046,358	460,000,058	151	383,482	929,129,994	2,422
*E*. *coli O157*:*H7*	3,794,862	548,505,079	144	403,919	1,100,295,861	2,724
*B*. *trehalosi*	1,718,212	249,216,010	145	205,096	499,939,066	2,437
*M*. *haemolytica*	1,724,414	249,368,724	144	175,953	531,234,319	3,019
*F*. *tularensis*	926,716	199,169,591	214	176,376	399,767,452	2,266
*S*. *enterica*	1,943,848	279,774,061	143	394,699	1,000,244,555	2,534
**Organism**	**Illumina MiSeq reads (25X)**			**PacBio RS reads (50X)**		
Mammal	329,484,322	62,120,890,467	188	17,695,174	146,961,409,902	8,305

The bacterial data are from 2013 and originate from the Sequencing Read Archive (SRA). The sequencing chemistries were not accurately described in the original datasets but the bacterial MiSeq read data represent either Illumina sequencing-by-synthesis chemistry v1 or v2. The mammal MiSeq read data are a mixture of Illumina sequencing-by-synthesis chemistry v2 and v3. The bacterial PacBio RS reads represent PacBio SMRT sequencing chemistries that are earlier than P4-C2 and the mammal PacBio RS reads are a mixture of PacBio SMRT sequencing chemistries P5-C3 and P6-C4.

**Table 2:** *De novo* assembly, scaffolding and gap filling statistics for the six bacterial draft genomes and the mammal draft genome.

		Num. sequences						
Organism	Tool	Expected	Final	Sum (bp)	N50 length	Gap #	Gap (bp)	Gap %
*E*. *coli K12 MG1655*	SPAdes	1	35	4 661 027	4 640 853	0	0	0,00%
* *	SSPACE-LR	1	34	4 661 028	4 641 005	1	1	0,00%
* *	gapFinisher	1	34	4 661 028	4 641 005	1	1	0,00%
* *	PBJelly	1	34	4 661 335	4 641 312	1	1	0,00%
* *	GMcloser	1	34	4 661 028	4 641 005	1	1	0,00%
* *	miniasm	1	1	4 793 967	4 793 967	0	0	0,00%
*E*. *coli O157*:*H7*	SPAdes	10	87	5 547 646	3 323 349	3	3	0,00%
* *	SSPACE-LR	10	50	5 568 199	3 323 349	29	18486	0,33%
* *	gapFinisher	10	50	5 568 974	3 323 349	13	5750	0,10%
* *	PBJelly	10	49	5 590 669	3 323 349	13	**5411**	0,10%
* *	GMcloser	10	50	5 568 199	3 323 349	28	18634	0,33%
* *	miniasm	10	25	5 898 494	537 223	0	0	0,00%
* *	SSPACE-LR	10	16	5 908 008	612 090	9	9514	0,16%
* *	gapFinisher	10	16	5 907 537	612 090	5	**2495**	0,04%
* *	PBJelly	10	16	5 925 577	612 150	5	3683	0,06%
* *	GMcloser	10	16	5 908 008	612 090	9	7392	0,13%
*B*. *trehalosi*	SPAdes	1	51	2 376 258	274 711	2	2	0,00%
* *	SSPACE-LR	1	12	2 401 287	438 635	13	2804	0,12%
* *	gapFinisher	1	12	2 401 265	438 599	5	401	0,02%
* *	PBJelly	1	11	2 412 762	1 257 245	**4**	**28**	0,00%
* *	GMcloser	1	12	2 401 287	438 635	13	2804	0,12%
* *	miniasm	1	17	2 510 680	221 473	0	0	0,00%
* *	SSPACE-LR	1	10	2 520 563	377 524	7	9883	0,39%
* *	gapFinisher	1	10	2 521 341	377 524	3	4920	0,20%
* *	PBJelly	1	10	2 529 003	378 677	**2**	**1887**	0,07%
* *	GMcloser	1	10	2 520 563	377 524	7	9539	0,38%
*M*. *haemolytica*	SPAdes	1	112	2 664 209	101 958	35	35	0,00%
* *	SSPACE-LR	1	17	2 718 326	1 073 880	80	13504	0,50%
* *	gapFinisher	1	17	2 717 906	1 073 740	**38**	4498	0,17%
* *	PBJelly	1	17	2 735 092	1 078 177	45	**1156**	0,04%
* *	GMcloser	1	17	2 718 326	1 073 880	69	13670	0,50%
* *	miniasm	1	10	2 926 783	378 549	0	0	0,00%
* *	SSPACE-LR	1	8	2 928 560	378 549	2	1777	0,06%
* *	gapFinisher	1	8	2 928 834	378 549	**1**	1155	0,04%
* *	PBJelly	1	8	2 935 736	380 443	2	**1110**	0,04%
* *	GMcloser	1	8	2 928 560	378 549	2	1674	0,06%
*F*. *tularensis*	SPAdes	3	135	1 807 729	25 688	0	0	0,00%
* *	SSPACE-LR	3	58	1 855 045	56 838	97	23176	1,25%
* *	gapFinisher	3	58	1 851 864	56 791	**24**	11254	0,61%
* *	PBJelly	3	28	1 892 167	380 120	28	**52**	0,41%
* *	GMcloser	3	58	2 173 335	63 394	125	24436	1,12%
* *	miniasm	3	20	2 000 228	15 305	0	0	0,00%
* *	SSPACE-LR	3	9	2 021 978	426 098	11	21750	1,08%
* *	gapFinisher	3	9	2 021 618	425 969	7	16843	0,83%
* *	PBJelly	3	9	2 033 224	425 957	**5**	**7416**	0,36%
* *	GMcloser	3	9	2 021 978	426 098	11	19947	0,99%
*S*. *enterica*	SPAdes	4	217	4 982 997	153 597	653	655	0,01%
* *	SSPACE-LR	4	94	5 026 381	1 020 795	723	10050	0,20%
* *	gapFinisher	4	94	5 028 882	1 020 937	644	2917	0,06%
* *	PBJelly	4	90	5 043 631	1 294 552	688	**765**	0,02%
* *	GMcloser	4	94	5 026 167	1 020 795	**620**	14759	0,29%
* *	miniasm	4	16	5 373 212	735 723	0	0	0,00%
* *	SSPACE-LR	4	10	5 384 667	874 322	6	11455	0,21%
* *	gapFinisher	4	10	5 384 057	874 322	**2**	4641	0,09%
* *	PBJelly	4	10	5 391 204	874 734	3	**2446**	0,05%
* *	GMcloser	4	10	5 384 667	874 322	6	11372	0,21%
*Mammal*	SPAdes	Unknown	236592	2 253 617 865	19 739	0	0	0,00%
	SSPACE-LR	Unknown	10143	2 462 623 627	599 108	42861	10136364	0,41%
	gapFinisher	Unknown	10143	2 466 785 189	601 444	**40427**	6945295	0,28%
	PBJelly	Unknown	9986	2 504 610 103	612 925	41059	**1703559**	0,07%
	GMcloser	Unknown	-	-	-	-	-	-
	miniasm	Unknown	1314	2 460 097 408	8 668 858	0	0	0,00%
	SSPACE-LR 1st run	Unknown	1115	2 460 626 045	9 381 548	199	528637	0,02%
	gapFinisher 1st run	Unknown	1115	2 460 674 964	9 381 548	**129**	351878	0,01%
	PBJelly 1st	Unknown	1115	2 462 063 881	9 385 435	152	**314033**	0,01%
	GMcloser 1st	Unknown	-	-	-	-	-	-
	SSPACE-LR 2nd 2nd run	Unknown	1008	2 460 965 525	9 562 827	236	642439	0,03%
	gapFinisher 2nd run	Unknown	1008	2 460 993 827	9 562 827	218	616617	0,03%
	PBJelly 2nd	Unknown	1008	2 461 884 336	9 562 882	**204**	**454681**	0,02%
	GMcloser 2nd run	Unknown	-	-	-	-	-	-

For clarity, only the most meaningful results of the benchmark are shown here. The full results are provided as supporting information and the assembly workflows are also better visualized there (S1 Table). The best gap filling results for each of the draft genomes are presented as bold font figures (columns 7 and 8).

For the Illumina short reads, we further applied the Fast Length Adjustment of SHort Reads (FLASH) protocol that finds overlaps at the ends of the paired-end reads and joins the reads if found [29]. Thus, about half of the reads in each dataset could be combined to longer initial fragments before the contig assembly. This feature is likely to improve the *de novo* genome assemblies while longer initial read length may be enough to span short repeats, insertions and deletions. The uncombined reads from the FLASH protocol were supplied as additional paired-end libraries in all assemblies. The Roche 454 Genome Sequencer data available for the draft genomes was not utilized here, as our benchmark did not include a suitable assembler, e.g. Newbler [30] for these data. Furthermore, the performance of Newbler was extensively evaluated in the SSPACE-LongRead original publication and in most of the cases Newbler could not perform as well as the other short read assemblers [9].

**Fig 3:**
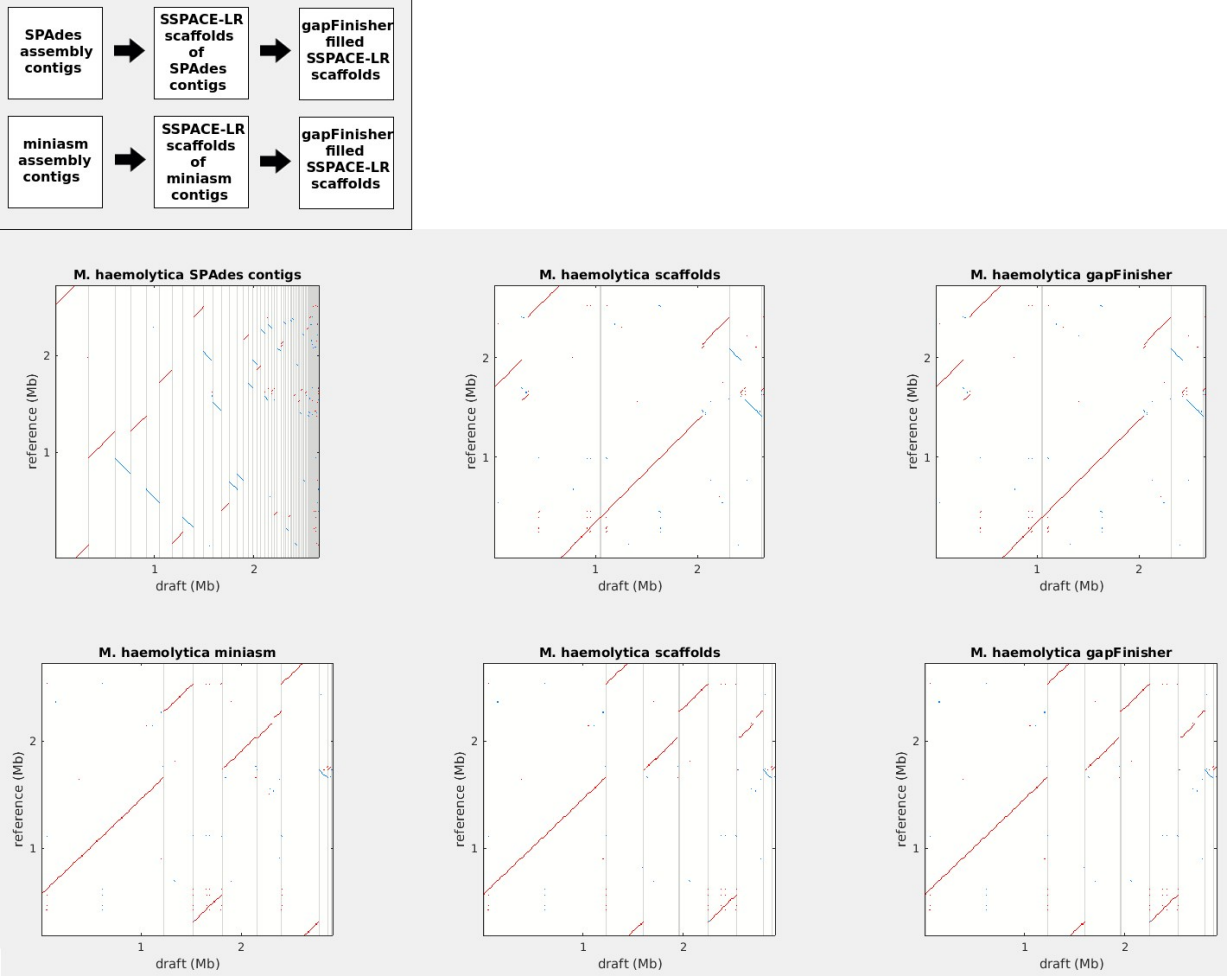
minidot [28] plots of the *Mannheimia haemolytica* draft genome at different stages of the assembly. **Upmost left:** Image key and reading direction. **Top left:** SPAdes contig assembly, **top center:** scaffold stage of SPAdes contigs, **top right:** gap filling stage, of the *M*. *haemolytica* draft genome. **Bottom left:** miniasm [28] contig assembly, **bottom center:** scaffold stage of miniasm contigs, **bottom right:** gap filling stage, of the *M*. *haemolytica* draft genome. The red diagonal lines indicate continuous regions of alignment between the draft assembly and the *M*. *haemolytica* reference sequence. The blue diagonal lines indicate regions with inverted alignment. The red and blue dots indicate repeats and inverted repeats, respectively. Draft assembly contig/scaffold boundaries are shown as grey vertical lines. The alignment plots are provided for each of the bacterial genomes (S1 Table).

We assembled the draft genomes with the SPAdes [31] and miniasm [28] assemblers. SPAdes can employ both Illumina short reads and PacBio CLR reads. In contrast, miniasm only works properly with PacBio CLR reads or other long reads with a sufficient sequencing coverage. This is because the read trimming phase of miniasm requires a read-to-read mapping length of at least 2,000 bp with a minimum of 100 bp non-redundant bases [28]. This condition is not met by the short-read datasets used in this study. An additional and a highly useful feature of miniasm is the minidot plot drawing utility and it was used to create the dotplots for comparisons to the reference genomes (Fig 3 and S1 Fig).

The bacterial initial assemblies were refined to scaffolds using PacBio filtered subreads. The scaffolding step included the combined use of SSPACE-LR (academic license, software version 1.1) [9] and the gapFinisher pipeline. We first executed SSPACE-LR for all samples to create the superscaffold assemblies for the six bacterial genomes and the unpublished *Phocidae* family mammal draft genome (Table 2). The same long read data was applied for the scaffolding of both SPAdes and miniasm contig assemblies. For each scaffold assembly, we then executed gapFinisher, PBJelly [6] and GMcloser [7] to fill the gaps introduced by the scaffolding step. Due to the large size (~2.5 gigabases) of the unpublished mammal genome, the SSPACE-LR and gap filling stage for the miniasm assembly had to be executed in two consecutive runs with 25X (50% of the total coverage) PacBio reads applied to each part. On the other hand, the scaffolding of the mammal SPAdes assembly was executed in five separate stages as part of the actual genome project of the mammal. About 10X coverage of PacBio reads of insert were applied at each stage and gapFinisher executed between the stages. Reads of insert are PacBio reads that have been self-corrected by aligning CLR reads (= filtered subreads) from the same molecule against themselves, a protocol originally described by Koren and coworkers [32]. This helps to filter out possible random sequencing errors in the long-read data with the expense of losing some of the read coverage in the process. The results for this assembly show statistics for the final stage and average CLR reads per scaffold is the average of all five stages (Table 2 and S1 Table).

**Fig 4:**
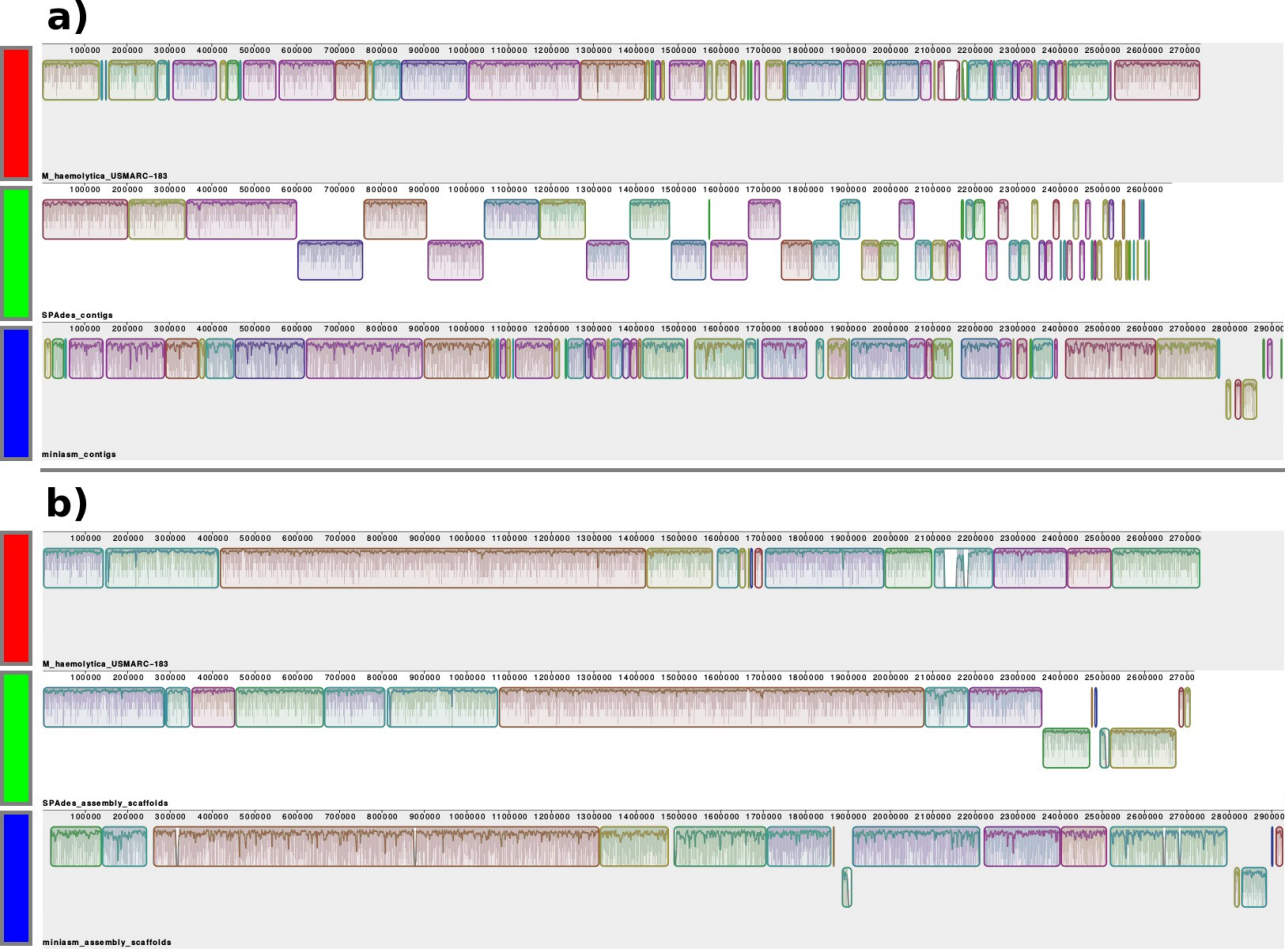
Mauve [33] alignments of the *Mannheimia haemolytica* genome. The visualizations are from **a)** before and **b)** after the scaffolding/gap filling stage. The corresponding Locally Collinear Blocks (LCB) in the three genome versions are indicated by different colors of horizontal bars. The darker lines within the blocks indicate local median similarity while the light lines show the range of local similarity values. White areas indicate low or no similarity. Blocks below the center line indicate regions that align in the reverse complement (inverse) orientation. **a):** *M*. *haemolytica* reference sequence (red bar), SPAdes [31] assembly contig sequences (green bar), and miniasm [28] assembly contig sequences (blue bar). **b):** *M*. *haemolytica* reference sequence (red bar), and gap filled scaffolds using the SPAdes assembly contig sequences (green bar), and the miniasm assembly contig sequences (blue bar).

We visualized the different stages of the draft assemblies for all genomes by compiling the minidot plots with the subplot utility of the MATLAB toolkit (Fig 3 and S1 Fig). Furthermore, we visualized the final stages of the assembly and scaffolding by aligning the reference genomes and the two drafts from the SPAdes and miniasm assembly pipelines with the progressiveMauve algorithm of the Mauve [33] alignment and visualization tool (Fig 4 and S2 Fig). Mauve reveals the number and similarity of Locally Collinear Blocks (LCBs) between the input sequences.

To assess the performance of the software, the SPAdes, miniasm, SSPACE-LongRead and the gap filling runs were executed in two separate 64-bit Linux computer environments. First, the bacterial genomes were assembled, scaffolded and gap filled in a single-processor (4 cores) computer running Ubuntu Linux 14.04 with 20 GB of RAM, the equivalent to a modern office workstation with a RAM extension. The 4-core processor was an Intel Core with a frequency of 3.2 GHz. Second, we built the mammal genome in a multi-core supercomputer running Ubuntu Linux 14.04 with 1 TB of RAM and using 16 Advanced Micro Devices Opteron processing cores with a frequency of 2.5 GHz each. The latter setup is equivalent to a small-scale local computer cluster. We used a built-in UNIX/Linux utility (/usr/bin/time) to measure the peak RAM use and elapsed computation times during each of the assembly stages.

We compared gapFinisher, PBJelly [6] and GMcloser [7] in the gap filling stage of the scaffolded SPAdes and miniasm assemblies. The PBJelly results are reported for all the six stages of the pipeline. With PBJelly, we decided to use 4 processing cores in the BLASR alignment step in the single-processor runs of the bacterial assemblies, as this step of the pipeline was expected to take an infeasibly long time otherwise. For GMcloser, the results are reported after three iterations of the tool with the same data that is the default setting.

## Results

**Fig 5:**
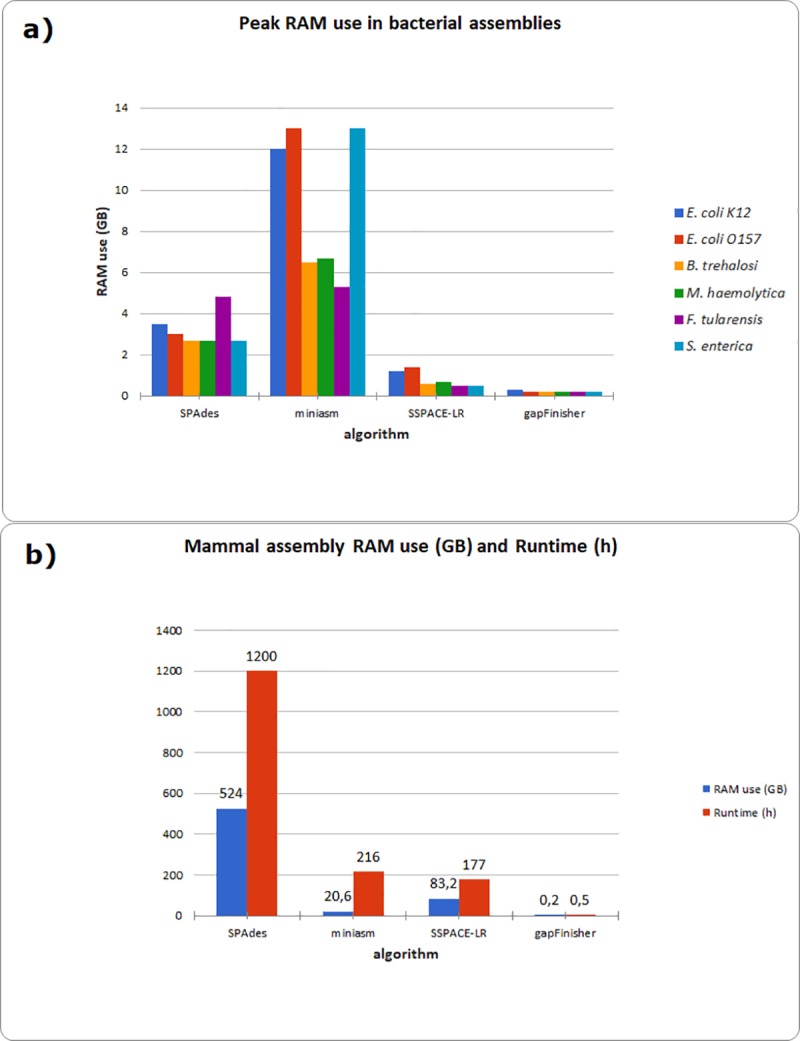
Performance benchmark of the assembly, scaffolding and gap filling tools used. The exact values are reported in S1 Table. **a)** Peak random access memory (RAM) use in gigabytes (GB) in the six bacterial assemblies. **b)** Peak RAM use (GB) and runtimes (in hours) of the assembly, scaffolding and gap filling algorithms in the marine mammal (*Phocidae* family) genome assembly.

**Fig 6:**
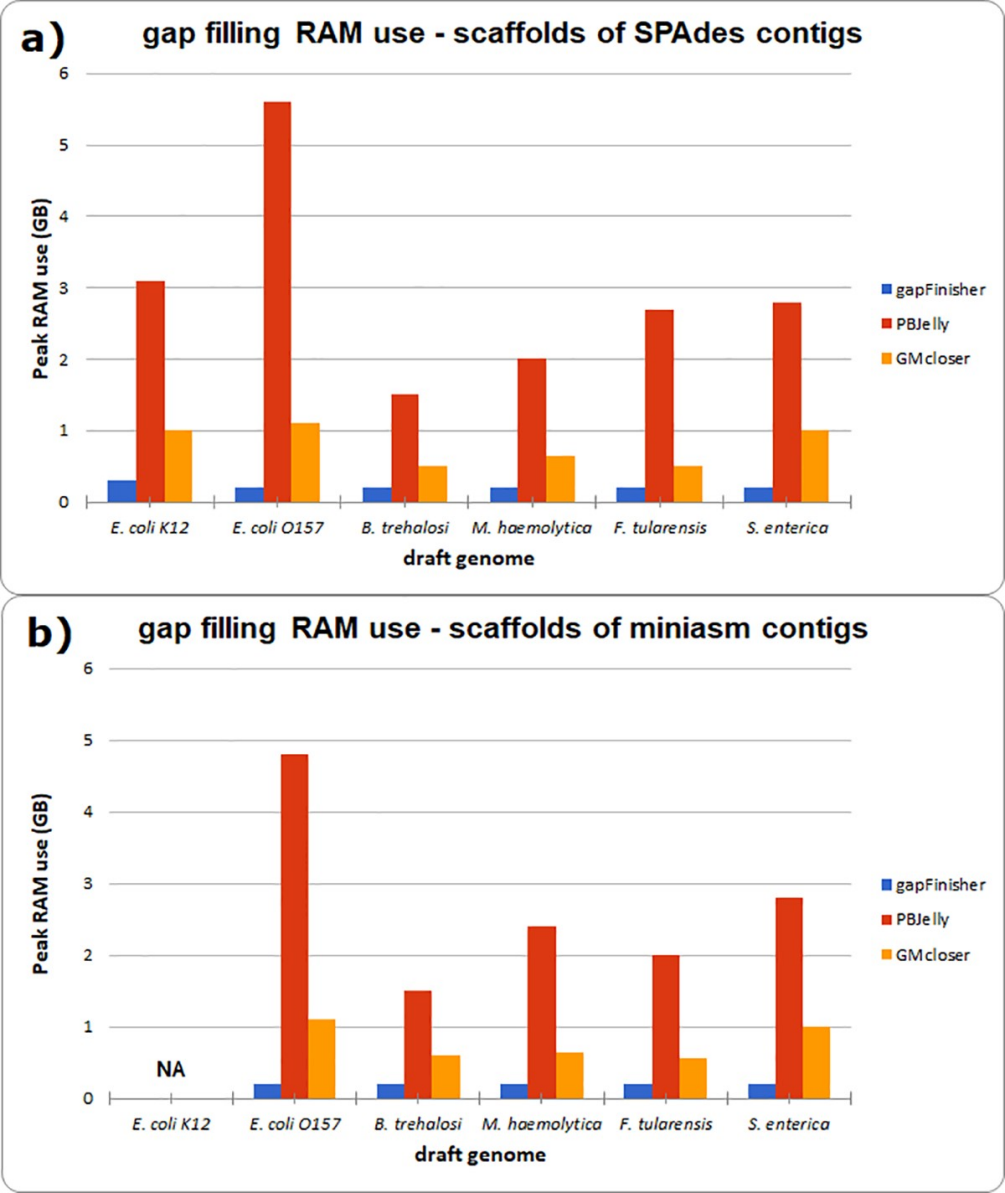
Gap filling peak RAM use of the bacterial assemblies with gapFinisher, PBJelly and GMcloser. **a)** Peak RAM use (as reported by the UNIX/Linux /usr/bin/time utilitity) of the SPAdes assembly scaffolds. **b)** Peak RAM use (as reported by the UNIX/Linux /usr/bin/time utilitity) of the miniasm assembly scaffolds. The RAM use data for *E*.*coli* K12-strain are missing (‘NA’) in b) due to the genome being closed to a single sequence with no gaps after the miniasm assembly.

**Fig 7:**
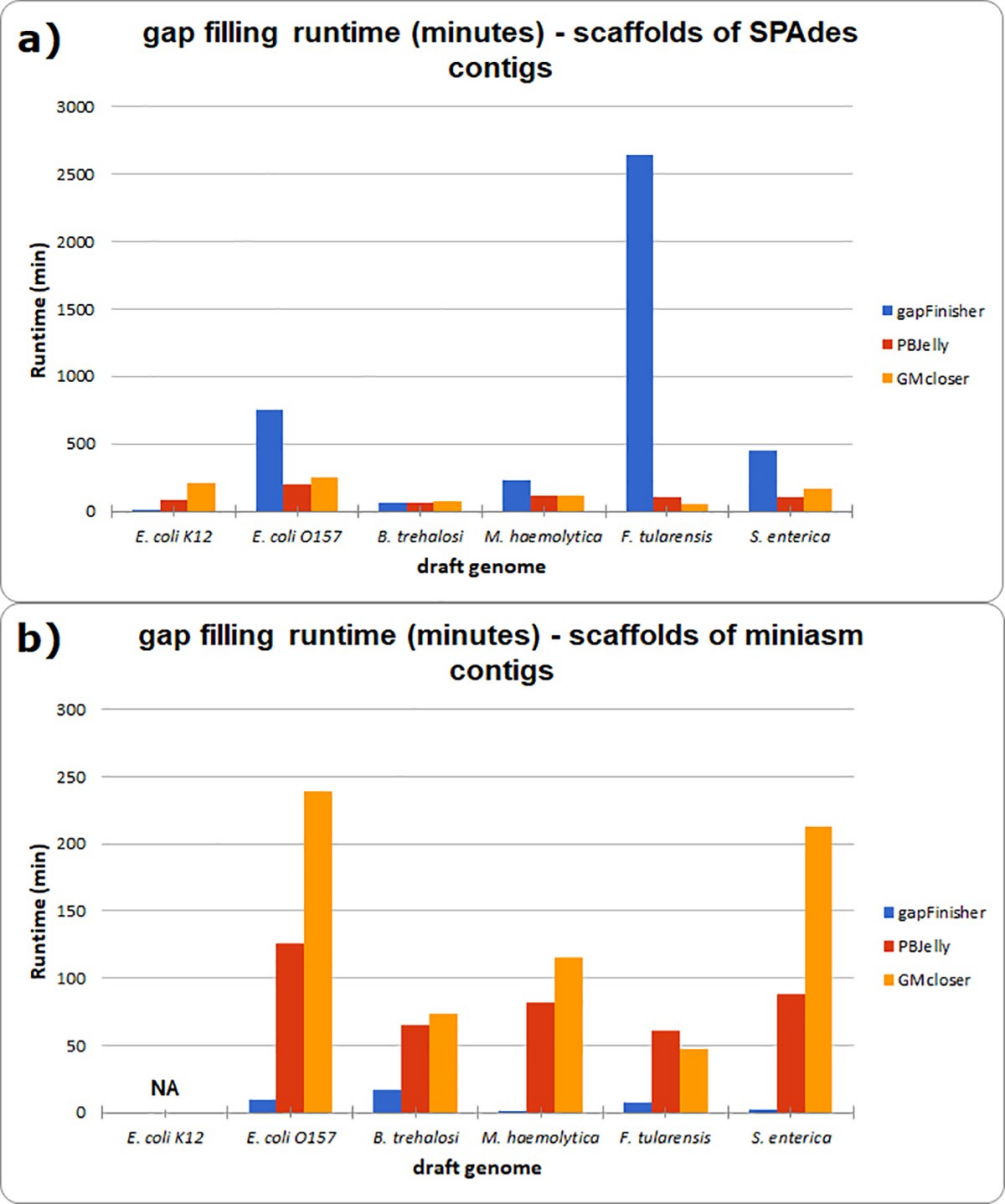
Gap filling runtimes of the bacterial assemblies with gapFinisher, PBJelly and GMcloser. **a)** Runtimes (in minutes) of the SPAdes assembly scaffolds. **b)** Runtimes (in minutes) of the miniasm assembly scaffolds. The runtimes for *E*.*coli* K12-strain are missing (‘NA’) in b) due to the genome being closed to a single sequence with no gaps after the miniasm assembly.

The results are presented both from the viewpoint of how finished the draft genomes were before and after the gap filling stage and how gapFinisher performed with respect to PBJelly [6] and GMcloser [7]. Key statistics of the assembly benchmark results were compiled (Figs 5, 6 and 7) and the alignments of gapFinisher-filled draft genomes to the bacterial reference genomes were visualized (Figs 3 and 4 and S1 and S2 Figs.). In the tabulation of the results (Table 2 and S1 Table), the **N50 length** statistic implies the contig length for which 50% of the total length of the draft assembly is in contigs greater than or equal to this length. This is a common and a robust statistic to describe the distribution of sequence lengths in the assembly.

### Genomes

Regarding the *de novo* assembly of the genomes, we noticed similar behaviour of the SPAdes assembler as reported by the authors of the SSPACE-LongRead [9]. Namely, that the SPAdes assembly pipeline introduced repeats at the ends of the contigs that evidently prohibited many CLR reads from aligning into the contig ends and thus the scaffold assembly is left with a higher number of uncombined sequences (Figs 3 and 4A and Table 2). Nevertheless, scaffolding with SSPACE-LongRead reduced the number of total sequences in all the assemblies. This was especially evident in the *Mannheimia haemolytica* draft genome, where SSPACE-LongRead reduced the number of sequences in the draft assembly from 112 to 17 (84.8% reduction). A notable increase in basic assembly statistics, such as the N50 contig length and number of sequences, was observed throughout (Table 2). The miniasm assembler [28] outperformed the assemblers used in the SSPACE-LR test assemblies [9] and the SPAdes assembler [31] in our benchmark in terms of the number of output contigs, N50 and gap length (Table 2). On the other hand, the median similarity of the alignments to the bacterial reference genomes is lower across all bacterial draft genomes from the miniasm pipeline (Fig 4B and S2 Fig).

For the *E*. *coli* K12 genome, the number of SPAdes assembly contigs was the lowest of the bacterial assemblies in this study, namely 35 (Table 2). The miniasm assembly of the *E*. *coli* K12 genome was a single sequence (Table 2 and S1 Table) and thus was the only draft genome not to require scaffolding or gap filling. Furthermore, miniasm was able to construct the full *E*. *coli* K12 genome from PacBio reads in 3 minutes (S1 Table). The final assembly consists of a single long bacterial genome (Table 2) contained in 4 Locally Collinear Blocks (LCB’s) according to progressiveMauve [32] alignment (S2 Fig and S1 Table). The contig assembly results for the other bacterial genomes were more variable with both SPAdes and miniasm (Table 2 and Figs 3 and 4).

Of the assemblers included in our benchmark, miniasm consistently reports zero N’s at the contig assembly stage (Table 2). Furthermore, the miniasm contig assemblies are more contiguous in the sense that they consist of less sequences when compared to the SPAdes assemblies in all cases (Table 2). This also means that the miniasm contigs are longer than SPAdes contigs. However, the SPAdes contig assemblies reported some gapped sequences with *E*. *coli O157* (3 bp), *B*. *trehalosi* (2 bp), *M*. *haemolytica* (35 bp) and *S*. *enterica* (655 bp) (Table 2).

Regarding the gap filling step, there was not a single tool that would have outperformed all the other approaches in all of the draft genomes we tested: gapFinisher reduced the number of N’s in all draft genomes. PBJelly generally performed better than gapFinisher and GMcloser in terms of the percentage of gaps filled, but in the case of both *M*. *haemolytica* assemblies, *F*. *tularensis* SPAdes assembly, *S*. *enterica* miniasm assembly and the mammal SPAdes assembly, gapFinisher filled numerically more gaps than PBJelly (Table 2). In the case of *E*. *coli* O157 SPAdes assembly, gapFinisher was the best gap filling tool also percent-wise. The GMcloser results in gap filling were poor: The scaffolded SPAdes assemblies in all the bacterial genomes showed that the number of gapped sequence (N’s) in the genome stayed the same or often increased after GMcloser (Table 2). In the miniasm assemblies 1–5% of gaps were filled by GMcloser, a notably lower rate than with gapFinisher and PBJelly (50% or more). GMcloser was able to numerically reduce more gaps than gapFinisher and PBJelly only in the case of *S*. *enterica* SPAdes assembly, but even there the number of gapped sequence increased from 0.20% to 0.26% of the total length of the assembly (Table 2). The GMcloser run for the draft mammal genome SPAdes assembly was aborted after it had not finished the first of the default three iterations in 3122 hrs (ca. 130 days). GMcloser runs were discontinued to the rest of the mammal genome drafts after this. The performance of PBJelly was outstanding also in the mammal genome assemblies. This was especially evident in the SPAdes assembly, where PBJelly reduced the number of gapped sequence by 83.2% (Table 2). The results also show that in 5 of the 14 assemblies, the final number of sequences in the draft genome was decreased after PBJelly, which means that PBJelly performs additional scaffolding where possible. GMcloser and gapFinisher do not have this feature.

Evidently, gapFinisher could fill about 50% of the gapped sequence (Table 2) in the scaffolded draft genomes and retained the structure of the genomes in all cases (Figs 3 and 4 and S1 and S2 Figs). The lowest percentage of gaps filled was with the second stage of the mammal genome miniasm scaffolding (4.1%) and the highest percentage of gaps filled was with the scaffolding of the *B*. *trehalosi* SPAdes assembly (85.7%). At the nucleotide level, several kilobases of gapped sequence was filled in all draft genomes (Table 2). No large insertions, deletions or inversions were introduced by the gap filling stage with gapFinisher (Table 2 and Fig 3 and S1 Fig). There were no cases of gapFinisher warning about separate reads from the same SMRT cell well attempting to fill disparate gaps in any of the bacterial genomes.

### Performance

The gapFinisher pipeline is easier to use compared to PBJelly[6] and GMcloser[7]: Besides MATLAB Compiler Runtime and a Perl [17] interpreter, gapFinisher does not require any other software to be installed. Furthermore, the gapFinisher pipeline is contained in a single phase, namely the actual execution of the gap filling, where e.g. the PBJelly [6] pipeline has six separate phases.

Due to the serial design of the pipeline, gapFinisher runtime holds quite neatly at about 3–5 wall-clock seconds per CLR read per scaffold (S1 Table and Fig 7). Thus, gapFinisher computation times prove linear with relation to the number of input scaffolds and the total coverage of the long reads that participated in the scaffolding. Where the average number of CLR reads per created scaffold was high, as was the case with the SPAdes-assembled bacterial genomes of *E*. *coli* O157:H7-strain, *F*. *tularensis*, *M*. *haemolytica* and *S*. *enterica*, gapFinisher running time in single-core mode was notably higher (Fig 7A and S1 Table).

Nevertheless, gapFinisher generally runs quicker than the other tested gap filling tools even in a single-processor, single-core, setting. In the gap filling of the miniasm assemblies, runtimes were clearly highest for GMcloser (Fig 7B, S1 Table). It further looks like that GMcloser is not scalable to larger genome: The benchmark run for the draft mammal genome had to be aborted after it had not finished in a reasonable time (S1 Table).

We studied the random access memory (RAM) use of gapFinisher (Fig 5) and compared this with the other gap filling tools (Fig 6 and S1 Table). Again, the serial design of gapFinisher keeps the RAM use of the gap filling stage at all but nominal level (Figs 5 and 6). This feature applies also to the gap filling of the much larger mammal genome (Fig 5B and S1 Table). In general, the peak RAM use of less than 1 GB we detected in all cases means that gapFinisher could be executed for any genome in almost any Linux computer, even most laptops and tablets. Nevertheless, the preceding assembly steps tend to use significantly more RAM (Fig 5B). The larger mammal genome used more than 500 GB of RAM in the contig assembly stage and more than 80 GB of RAM in the SSPACE-LongRead stage (Fig 5B and S1 Table).

## Discussion

Gap filling is a non-trivial problem with many existing solutions today in the form of software tools. The correctness of the outputs of different tools is variable. For a large genome under assembly, the default parameter settings of FGAP [8] clearly are too lenient and may lead to incorrect gap filling in large draft genomes (S2 Table). Repeat masking before the gap filling step could be recommended [22], especially because FGAP utilizes BLAST [14] directly for the long-read alignment.

Typically, contig assemblies do not contain unknown sequences (N-characters) and the output of miniasm correctly follows this principle (Table 2). However, it is evident from the SPAdes assembler results that a small number of N’s may be introduced already at the contig assembly stage (Table 2). This may be due to the N’s present in the sequencing read data that is not uncommon for Illumina sequencing reads but is more unusual for PacBio long reads. Our results indicate that both the SPAdes and miniasm assemblers are optimized for the *E*. *coli* K12 genome: The number of *E*. *coli* K12 SPAdes assembly contigs was the lowest of the bacterial assemblies (Table 2) and the the *E*. *coli* K12 genome miniasm assembly was closed to a single sequence with no need for scaffolding or gap filling (Table 2 and S1 Table). Moreover, the *E*. *coli* K12 SPAdes assembly N50 length is close to the total size of the assembly (Table 2). This indicates an skewed contig length distribution of the assembly. A closer inspection of the 35 contigs showed one ca. 4.64 Mb contig and 34 low-complexity contigs with lengths between 128 and 2,553 bases (sequences not shown here). The 4.64 Mb contig shows high similarity to the whole *E*. *coli* K12 reference genome, as evident from the alignment dotplot against the reference (S1 Fig, subfigure a), and the length of the contig is 99,98% of the reference genome length (Table 2).

gapFinisher is not able to fill all gapped sequences in the draft assembly (Table 2). This is because the CLR reads of the Pacific Biosciences platform do contain base-call errors [11] and gapFinisher employs a strict alignment scheme of the long reads and only fills a gap when a reasonably correct alignment of known sequences at the gap edges is found (Figs 1C and 2). Consequently, some gaps may be prevented from filling, lacking sufficient evidence. A solution is to run gapFinisher on less strict parameters and then confirm the correctness of the result using other alignment tools. Nevertheless, gapFinisher with the default settings can reduce the amount of gapped sequence in the example draft genomes by about 50% in general (Table 2). However, in terms of RAM use, gapFinisher clearly outperformed PBJelly and GMcloser, the two other gap filling tools included in the test benchmark of this study (Fig 6). This was especially true for the large mammal draft genome (Fig 5B and S1 Table). It is likely that repetitive sequences in the ends of SPAdes contigs confused the workflow of GMcloser. The result was the increased amount of gapped sequence in the final scaffolds in most of the scaffolded SPAdes assemblies (Table 2 and S1 Table).

Regarding the use of filtered subreads in the bacterial genome assemblies of this study, gapFinisher did not detect any cases where separate reads from the same SMRT cell well would have filled disparate gaps in the genomes. In applications where conflicting read origins could be a problem, it can be circumvented by producing reads of insert from the filtered subreads with the expense of genome level read coverage. On the other hand, the reads of insert pipeline improves the overall quality of the reads which leads to more reliable alignments. Checking the read origin of the filtered subreads is a valuable additional correctness feature of the gapFinisher pipeline not available in the other gap filling tools presented in this study.

We found that the runtimes of gapFinisher are approximately linear with respect to the number of input scaffolds and the number of long reads related to each of the gaps in the scaffolds (Fig 5 and S1 Table). While the peak RAM use of gapFinisher stays at a nominal level for small and large genomes (Fig 5A and Fig 5C), the runtime varies significantly, even in small genome assemblies (Fig 5B). This feature will be optimized in the future development versions of gapFinisher. If the user can run gapFinisher in a computer with multiple cores, it is possible to specify the number of threads (option -t). Consequently, gapFinisher will divide the input scaffolds into even parts, splitting the total running time of the pipeline by the number of processors assigned. The parallelization would have significantly reduced the runtime in the gap filling of the SPAdes-assembled bacterial genomes of *E*. *coli* O157:H7-strain, *M*. *haemolytica* and *S*. *enterica* (Fig 5B and S1 Table). The effects of parallelization could be clearly seen in the case of the mammal genome gap filling where gapFinisher performed the gap filling task in ca. 30 minutes for all the drafts (Fig 5B), PBJelly took more than 100 hours and GMcloser was unable to finish in reasonable time (S1 Table).

No matter which next-generation sequencing platform is in use, base-call error profiles do affect the output and the quality of the sequenced reads. Previously, sequence-specific systematic miscalls have been reported in the output of Illumina Genome Analyzer II platform) [12, 34]. Evidently, the more recent Illumina MiSeq platform is affected by the same miscall profile to some extent [13, 35]. The presence of a relatively high error-rate can also not be disputed in current high-throughput sequencing of long reads [11]. High error-rate is also a likely explanation to the observed lower overall similarity of locally collinear blocks (LCBs) in the alignment of the genomes assembled with long-reads in miniasm (Fig 4 and S2 Fig). Nevertheless, with ever-improving sequencing chemistries and throughput the issue of high error-rates is likely to grow smaller in the future. Error profile aware quality control methods could also help to counter the various miscalls and other artefactual errors produced by most NGS platforms.

The sequencing coverage, and the length of the long-reads plays an important role in the finalization of the genomes: In the SSPACE-LR bacterial genome study, it was found that PacBio coverage from around 60X upwards did not further improve genome closure on the contig level [9]. Regarding read error-rates, it is already possible to self-correct PacBio CLR reads by using the reads of insert pipeline of the SMRT Analysis toolkit. For each sequenced molecule, an improved consensus sequence is obtained by aligning all the produced subreads together which cancels out the random errors in individual reads. The final quality of the sequence depends on the number of subreads obtained for each single molecule. Thanks to the nearly random error profile of the PacBio RS II instrument, single nucleotide miscalls in the reads will not be propagated to the reads of insert output, that is, the circular consensus (CCS) reads. Furthermore, the new Sequel instrument of Pacific Biosciences reportedly has 7-fold throughput as compared to the earlier RS II platform. This has major ramifications also for the total throughput of corrected reads from the platform.

There may be additional approaches to the gap filling problem. In theory, a simple gap-tolerant alignment of sequencing reads of variable lengths using existing mapping tools would be able to reliably span at least short gaps, say 1–20 bp in length. This is one of the near-future prospects of solving the gap filling problem, especially as the average read lengths of next-generation sequencing platforms are likely to only increase in the future.

## Conclusions

Despite the recent developments in next-generation sequencing technologies, unknown sequences continue to exist in published draft assemblies of small and large genomes [5]. Here, we presented an automated pipeline to solve the gap filling problem using the output of SSPACE-LongRead [9] and FGAP [8] in a controlled manner and wrapping these methods together in a pipeline called gapFinisher. Our pipeline utilizes both masked and unmasked draft genomes with gaps and ensures the uniqueness of the BLAST alignments returned by the FGAP algorithm by iterating through the read data one read and one input scaffold at a time. The serial design of gapFinisher keeps the computational footprint at a nominal level (Table 2 and Figs 5B, 6 and 7). As evident from the result statistics (Table 2) and the visualizations of the draft genomes (S1 and S2 Figs), gapFinisher performs efficient and reliable gap filling. Compared to PBJelly and GMcloser, gapFinisher generally performs faster and always has a smaller Random Access Memory footprint (Fig 6 and S1 Table). The performance of gapFinisher scales up to a large mammal genome (Fig 5B and S1 Table).

The use of gapFinisher is currently limited to SSPACE-LongRead academic license version output and requires the user to be able to run SSPACE-LongRead at least once. Nevertheless, SSPACE-LongRead currently is the only publicly available scaffolding software that can produce information about the sequences spanning the gaps in the final scaffolds. Should other utilities with this key feature become available, we will further develop gapFinisher for full compatibility. Our pipeline contributes to filling long gaps and solving the gap filling problem after scaffolding draft genomes of multiple organisms. While no present application can solve the gaps completely in the draft genomes, gapFinisher contributes to the gap filling step of both prokaryote and eukaryote genomes, even in published genome assemblies.

The read datasets for the bacterial genomes used in this study are available at: http://www.cbcb.umd.edu/software/PBcR/closure/index.html. The gapFinisher script to run the pipeline is made public under GNU’s general public license (GPL) version 3.0 and the binary distributions of the bundled utilities according to their specified licenses. gapFinisher can be downloaded at: http://www.github.com/kammoji/gapFinisher

## Supporting information

S1 Fig minidot [28] plots of the six bacterial genomes at different stages of the assembly. **a)** *E*. *coli* K12, **b)** *E*. *coli* O157:H7, **c)** *B*. *trehalosi*, **d)** *M*. *haemolytica*, **e)** *F*. *tularensis*, **f)** *S*. *enterica*. **Top left:** Image key and reading direction. **Top row** (in all subfigures): SPAdes [31] contig assembly, scaffolding and gap filling (gapFinisher) stages of the assembly. **Bottom row** (in all subfigures): miniasm [28] contig assembly, scaffolding and gap filling (gapFinisher) stages of the assembly. The scaffolding and gap filling stages are missing for the *E*. *coli* K12 assembly (**a)**) since the genome was in a single sequence (i.e. closed) after miniasm.
(PNG)

S2 Fig Mauve [33] alignments of the six bacterial genomes at different stages of the assembly. **a)** *E*. *coli* K12, **b)** *E*. *coli* O157:H7, **c)** *B*. *trehalosi*, **d)** *M*. *haemolytica*, **e)** *F*. *tularensis*, **f)** *S*. *enterica*. **Top part** (in all subfigures): progressiveMauve alignment of the respective bacterial reference genome (red bar), the SPAdes [31] contig draft genome (green bar) and the miniasm (Li, 2016) contig draft genome (blue bar). **Bottom part** (in all subfigures): progressiveMauve alignment of the respective bacterial reference genome (red bar), the SPAdes assembly pipeline gap filled (gapFinisher) scaffolds (green bar) and the miniasm assembly pipeline gap filled (gapFinisher) scaffolds (blue bar). Only the contig assembly stage (top part) is shown for the *E*. *coli* K12 assembly (subfigure **a)**) since the genome had no gaps after miniasm.
(PNG)

S1 Table All de novo assembly, scaffolding and gap filling statistics for the six bacterial draft genomes and the mammal draft genome. In addition, the performance benchmark statistics are included in the last three columns.
(XLSX)

S2 Table Gap filling data used and FGAP [8] default test results reported for an unpublished draft genome of a marine mammal from the Phocidae family. An admittedly small number of Pacific Biosciences RS II platform circular consensus reads (*reads of insert*) with summed length of about 280 kbp filled 45.5 million unknown bases in the draft genome, a result reported by FGAP with the default alignment settings. By changing the FGAP command line options, one can adjust the number of BLAST [14] hits returned. By default, this is 200 hits. We ran another test, where we reduced this amount to 2 so that only the best two BLAST hits would be considered in the gap filling. Still, more than 4.5 million N’s were reportedly filled with our test set, a far greater number of bases than contained by the original read data used. The default BLAST alignment parameters of FGAP for opening and extending a gap are both set to the value 1. The default values in command line applications of BLAST for opening and extending a gap are set as 5 and 2, respectively, as written in the BLAST user guide [36]. Depending on the total score of the alignment, gap opening in the alignment is up to 80% and gap extension up to 50% more likely than the BLAST defaults. The minimum raw score of a BLAST hit in FGAP is set to value 25, a typical raw score value of highly dissimilar sequences irrespective of the gap penalty parameters. Moreover, a maximum of 200 BLAST results may be returned for a 70 percent identity in alignment length of 300 bp by default. In general, the default parameters of FGAP appear too lenient and may fill gaps based on alignments that are incorrect and may appear multiple times where unique alignments are desired.
(XLSX)
