# Supplementary figures and images for: gapFinisher: A reliable gap filling pipeline for SSPACE-LongRead scaffolder output
Source: PLoS One. 2019 Sep 9;14(9):e0216885. doi: 10.1371/journal.pone.0216885 (PMC6733440; doi:10.1371/journal.pone.0216885)

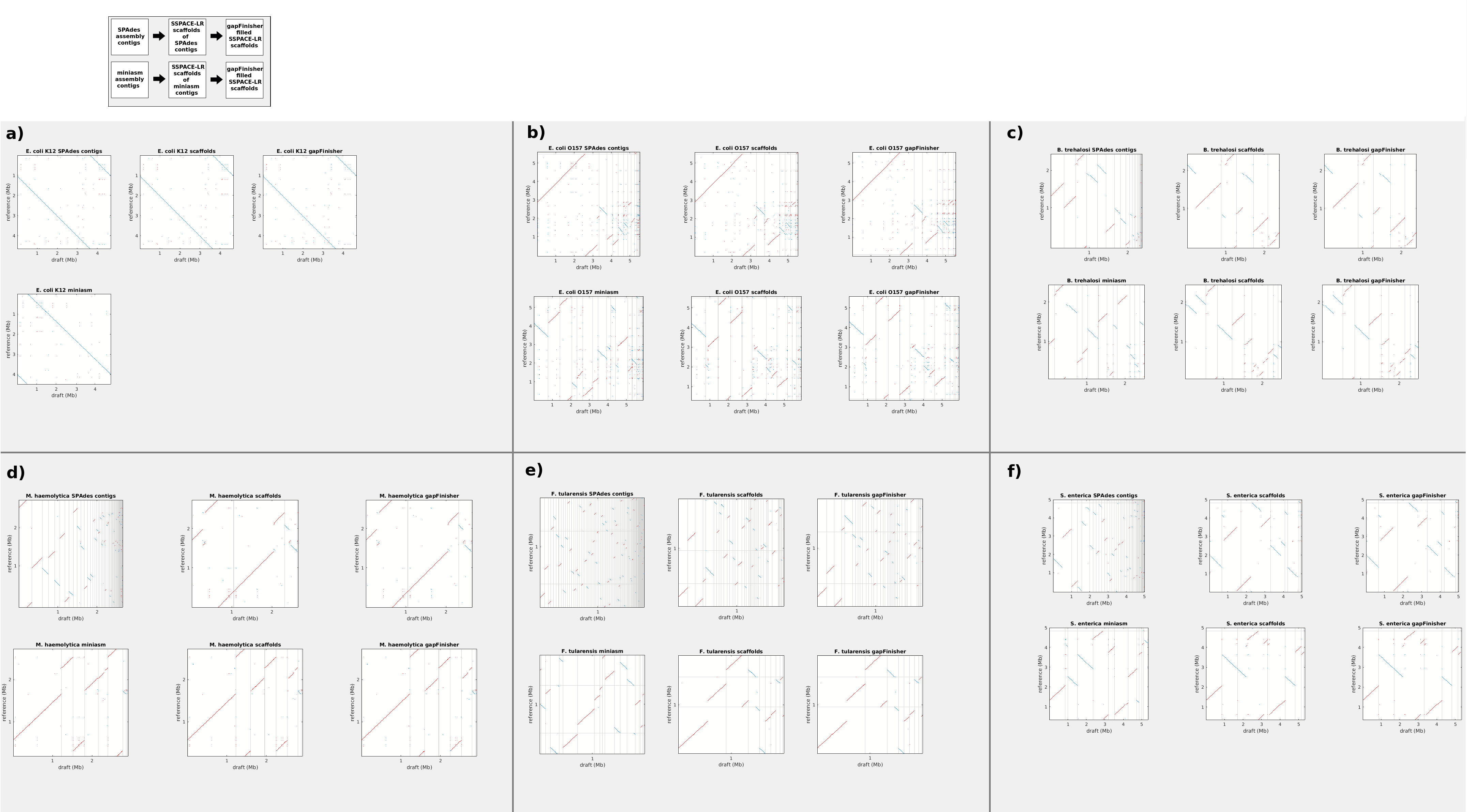

Supplement: S1 Fig — a) E. coli K12, b) E. coli O157:H7, c) B. trehalosi, d) M. haemolytica, e) F. tularensis, f) S. enterica. Top left: Image key and reading direction. Top row (in all subfigures): SPAdes [31] contig assembly, scaffolding and gap filling (gapFinisher) stages of the assembly. Bottom row (in all subfigures): miniasm [28] contig assembly, scaffolding and gap filling (gapFinisher) stages of the assembly. The scaffolding and gap filling stages are missing for the E. coli K12 assembly (a)) since the genome was in a single sequence (i.e. closed) after miniasm. (PNG) [file pone.0216885.s001.png]

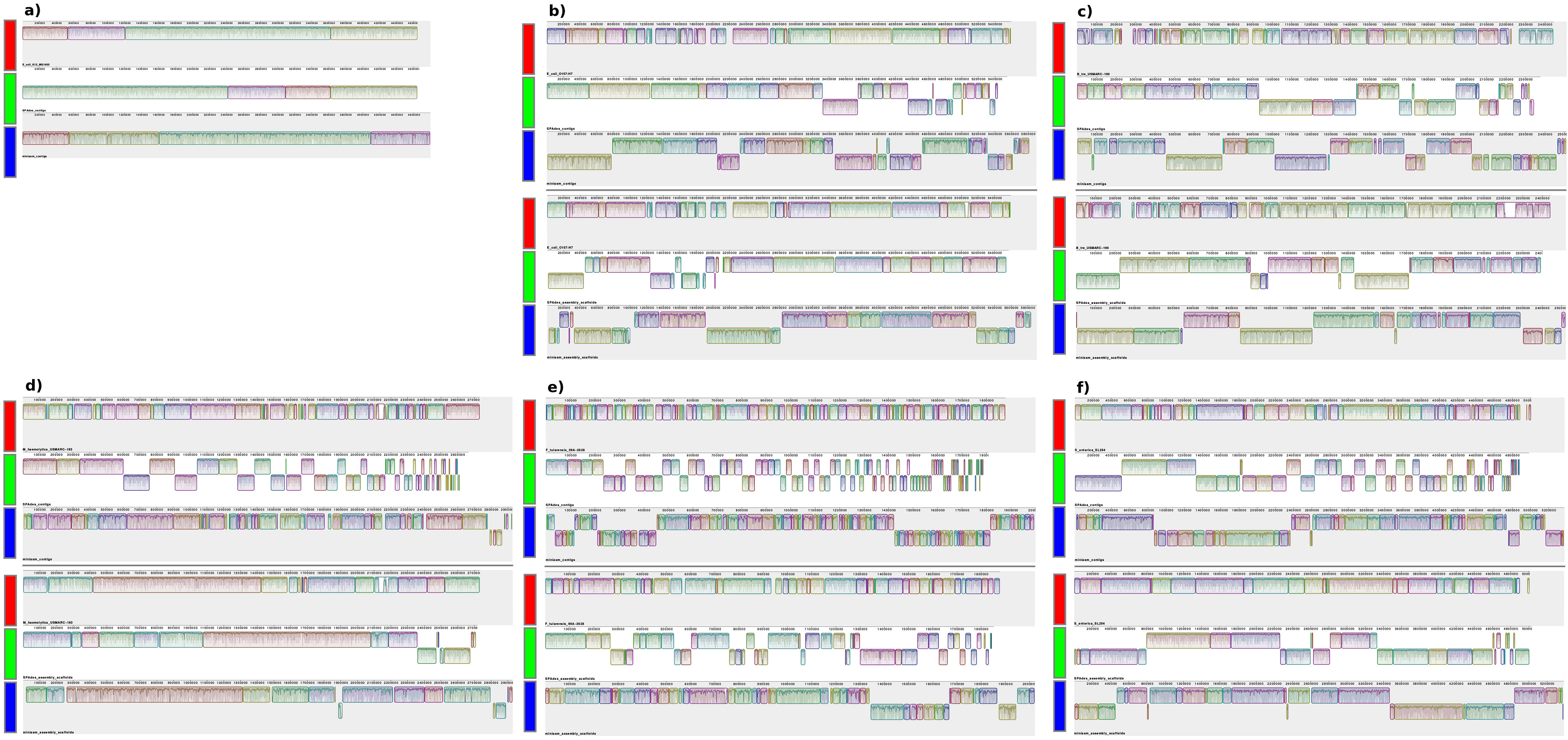

Supplement: S2 Fig — a) E. coli K12, b) E. coli O157:H7, c) B. trehalosi, d) M. haemolytica, e) F. tularensis, f) S. enterica. Top part (in all subfigures): progressiveMauve alignment of the respective bacterial reference genome (red bar), the SPAdes [31] contig draft genome (green bar) and the miniasm (Li, 2016) contig draft genome (blue bar). Bottom part (in all subfigures): progressiveMauve alignment of the respective bacterial reference genome (red bar), the SPAdes assembly pipeline gap filled (gapFinisher) scaffolds (green bar) and the miniasm assembly pipeline gap filled (gapFinisher) scaffolds (blue bar). Only the contig assembly stage (top part) is shown for the E. coli K12 assembly (subfigure a)) since the genome had no gaps after miniasm. (PNG) [file pone.0216885.s002.png]
